# Supplementary material for: Magnitude and associated factors of peripheral cytopenia among HIV-infected children attending at University of Gondar Specialized Referral Hospital, Northwest Ethiopia
Source: PLoS One. 2021 Mar 2;16(3):e0247878. doi: 10.1371/journal.pone.0247878 (PMC7924809; doi:10.1371/journal.pone.0247878)
Supplement: S1 File — (DOCX) [file pone.0247878.s001.docx]

**Annex III: Questionnaire (English version)**

Dear respondents, you are kindly requested to give correct information accordingly.

Thank you for your time and participation.

Participants code number_________________

**Part-I: Socio-demographic characteristics of children**

1. Children’s gender Male ⬜ Female ⬜
2. Children’s age: _________
3. Children educational status: No formal education ⬜ Primary School ⬜ secondary ⬜
4. Residence: Rural ⬜ Urban ⬜

**Part –II Socioeconomic /demographic characteristics of family/caregiver.**

1. Age of care giver: -________________
2. Gender: - Male ⬜ Female ⬜
3. Marital status: - Single ⬜ Married ⬜ Separated ⬜ Widowed ⬜ Divorced ⬜
4. Family occupation: - Privately employed ⬜ Gov’t employee ⬜ Merchant ⬜

House wife⬜ other_____

1. Family educational status: - No formal education ⬜ Primary School ⬜

Secondary school ⬜ College/ University ⬜

1. Parental status: - Both live ⬜ Both dead ⬜ Mother dead ⬜ Father dead ⬜
2. Relationship to child: Mother ⬜ Female caregiver⬜ Father ⬜ Male caregiver ⬜ other ­­­­_______
3. The HIV status of care giver: - positive ⬜ negative ⬜ not known ⬜
4. Family size: No__________________________
5. Family monthly income: - _________________________ Birr per month

**Part III -Children’s food habit**

1. Have you eaten dark green leafy vegetables like cassava, kale, spinach, cabbage?

Never ⬜ per day _____ per week______ per month________

1. Have eat meat and milk product (Beef, lamb, goat, chicken or other organ meats, milk, cheese, yogurt or other milk products)?

Never ⬜ per day _____ per week______ per month________

**PART- IV: -Clinical and anthropometric characteristics**

1. What was his/her WHO clinical staging? I ⬜ II ⬜ III ⬜ IV ⬜
2. Is there any opportunistic infection? Yes ⬜ No ⬜
3. If so, yes what type of infection? ________________
4. Your HAART exposure status? HAART Naïve ⬜ HAART experienced ⬜
5. If HAART experienced, date of treatment initiated (DD_______/MM______YY______
6. Current regimen type? _______________________________and ______________
7. HAART discontinue Yes ⬜ No ⬜
8. If so “yes” time: - __________________months
9. Is he/s being on second line ART treatment: - Yes ⬜ No ⬜
10. Type of second line ART treatment: ___________________________
11. Medication other than ART and prophylaxis: - Yes ⬜ No ⬜
12. If “yes” type of medication ____________________and ____________________
13. Is the children develop diarrhea? Yes ⬜ No ⬜
14. If so yes, type of diarrhea Acute ⬜ Chronic ⬜
15. Weight ___________ Kg 16. Height___________ Meter

**I thank you!!!**

**Annex VI: Questionnaire (Amharic version)**

**መጠይቅ**

ውድ ተሳታፊ ቀጥሎ ያለውን መጠይቅ ለመሙላትና ቃል ለመስጠት ስለተባበሩን እናመሰግናለን፡፡

መለያ ቁጥር**_________________**

**ክፍል -1: የህፃኑ ማህበረሰባዊ ባህርያቶች (Socio-Demographic Characteristics)**

1. የህፃኑ ጾታ፡- ወንድ ⬜ ሴት ⬜
2. የህፃኑ ዕድሜ፡- _____________________
3. የህፃኑ የት/ት ደረጃ:- መደበኛ ት/ት ያለተማረ ⬜ አንደኛ ደረጃ ⬜ ሁለተኛ ደረጃ ⬜
4. የመኖሪያ ቦታ፡- ገጠር ⬜ ከተማ ⬜

**ክፍል፡-2 የቤተሰብ /አሳዳጊ ማህበረሰባዊ ና ኢኮኖሚያዊ ባህሪያቶች**

1. የቤተሰብ /አሳዳጊ ጾታ፡- ወንድ ⬜ ሴት ⬜
2. የቤተሰብ /አሳዳጊ ዕድሜ፡- _____________________
3. የቤተሰብ /አሳዳጊ የጋብቻ ሁኔታ፡-ያገባች⬜ ያላገባች ⬜ የተለያዩ⬜ የሞተባት⬜
4. የቤተሰብ/አሳዳጊ የስራ ዓይነት፡- የግል ተቀጣሪ ⬜ የመንግስት ተቀጣሪ ⬜ ነጋዴ ⬜ ሌላ_____
5. የቤትስብ/አሳዳጊ የት/ት ደረጃ ፡- መደበኛ ት/ት ያለተማረ ⬜ አንደኛ ደረጃ ⬜ ሁለተኛ ደረጃ ⬜ ኮሌጅ/ዩኒቨርሲቲ ⬜
6. የቤተሰብ ሁኔታ፡- እናት/አባት በህይወት አሉ ⬜ አባት ብቻ አለ ⬜ እናት ብቻ አለች ⬜ እናት/አባት በህይወት የሌሉ ⬜
7. ከህፃኑ ጋር ያላቸው የዝምድና ሁኔታ፡-እናት ⬜ የሴት አሳዳጊ ⬜ አባት ⬜ የወንድ አሳዳጊ ⬜
8. በደምወ ውስጥ HIV ቫይረስ አለ፡- አለ ⬜ ለም ⬜ አላውቀም ⬜
9. ቤት ውስጥ ያለ የቤተሰብ ብዛት______­
10. የቤተስብ/አሳዳጊ የወር ገቢ መጠን፡- ______ብር

**ክፍል፡-3 የህፃኑ የአመጋገብ ሁኔታ**

1. ለምን ያህል ጊዜ ቅጠላቅጠል(ጎመን፤ሰላጣ፤ካሮት፤ቆስጣ) ይመገባል፡

በቀን______ በሳምንት______በወር_____ አልጠቀምም ⬜

1. ለምን ያህል ጊዜ የእንሰሳት ተዋፅኦ (የከብትስጋ፣የበግስጋ፣የፍየል ስጋ፣የዶሮ ስጋ እነዲሁም ወተት፤አይብ፣እርጎ የመሳሰሉትን ይመገባል፡-

በቀን____ በሳምን____ በወር______ አልጠቀምም ⬜

**ክፍል 4፡- ከህክምናና ከልኬታ ጋር የተያያዙ ባህርያቶች**

1. በዓለም አቀፍ የጤና መስፈረት መሰረት የታማሚው የበሽታ ደረጃ፡- I ⬜ II ⬜ III ⬜ IV⬜
2. በአሁኑ ሰአት ከኤድስ በተጨማሪ ሌላ ጥገኛ በሽታ አለበት፡- አለ ⬜ የለም ⬜
3. በተራ ቁ2 መልስዎ አለ ከሆነ የታየበት ጥገኛ በሽታ ስም፡- _______________ እና ___________
4. ከዚህ በፊት የኤድስ መድሃኒት ይወስድ ነበር፤- አወ ⬜ የለም ⬜
5. መልስዎ አወ ከሆነ ፣መድሃኒት የጀመረበት፤ ቀን____ ወር______አመት _____
6. የሚጠቀመው የመድሃኒት አይነት ________________________
7. መድሃኒት አቐርጧል፤ አወ ⬜ የለም ⬜
8. መልስወ አወ ከሆነ ለምን ያህል ጊዜ፤ _________________
9. ሁለተኛ ደረጃ የኤድስ መድሃኒት ይወስዳል ፤ አወ ⬜ የለም ⬜
10. መልስወ አወ ከሆነ የሚወስደው ሁለተኛ ደረጃ መድሃኒት አይነት ____________________________________________
11. ከኤድስ መድሃኒት ውጭ አሁን የሚወስደው በሽታን የሚከላከል(prophylaxis) እነዲሁም ሌላ መድሃኒት አለ፤ አወ ⬜ የለም ⬜
12. መልስወ አወ ከሆነ አሁን የሚወስደው መድሃኒት አይነት ፤ _________________
13. ታማሚው ተቅማጥ አለበት አወ ⬜ የለበትም ⬜
14. መልስወ አወ ከሆነ ምን አይነት ነው፤ አጣዳፊ ⬜ አጣዳፊ ያልሆነ⬜
15. ክብደት፤ _________________ሜትር
16. ቁመት ፤ __________________ ኪ/ግ

**አመሰግናለሁ!!!!!!!!**
